# DNA damage repair in ovarian cancer: unlocking the heterogeneity

**DOI:** 10.1186/s13048-018-0424-x

**Published:** 2018-06-20

**Authors:** Mary Ellen Gee, Zahra Faraahi, Aiste McCormick, Richard J. Edmondson

**Affiliations:** 1 Division of Cancer Sciences, Faculty of Biology, Medicine and Health, University of Manchester, St Mary’s Hospital, Manchester, UK; 2 Department of Obstetrics and Gynaecology, Manchester Academic Health Science Centre, St Mary’s Hospital, Central Manchester NHS Foundation Trust, Manchester Academic Health Science Centre, Level 5, Research, Oxford Road, Manchester, UK; 3 Northern Institute for Cancer Research, Newcastle University, Framlington Place, Newcastle upon Tyne, NE2 4AD UK

**Keywords:** Non-homologous end joining, Homologous recombination, Nucleotide excision repair, Base excision repair, Mismatch repair, Ovarian cancer

## Abstract

Treatment for advanced ovarian cancer is rarely curative; three quarters of patients with advanced disease relapse and ultimately die with resistant disease. Improving patient outcomes will require the introduction of new treatments and better patient selection. Abrogations in the DNA damage response (DDR) may allow such stratifications.

A defective DNA-damage response (DDR) is a defining hallmark of high grade serous ovarian cancer (HGSOC). Indeed, current evidence indicates that all HGSOCs harbour a defect in at least one major DDR pathway. However, defective DDR is not mediated through a single mechanism but rather results from a variety of (epi)genetic lesions affecting one or more of the five major DNA repair pathways. Understanding the relationship between these pathways and how these are abrogated will be necessary in order to facilitate appropriate selection of both existing and novel agents.

Here we review the current understanding of the DDR with regard to ovarian, and particularly high grade serous, cancer, with reference to existing and emerging treatments as appropriate.

## Background

Epithelial ovarian cancer is not one homogenous disease, but a collection of disparate cancers arising from different tissues, including the fallopian tube and the ovary, each of which represents a very different biological entity. Current evidence suggests five major subtypes of epithelial ovarian cancer, namely, high grade serous, low grade serous, endometrioid, clear cell and mucinous cancers, of which high grade serous is the commonest.

Teasing out the heterogeneity between these types is of major importance but remains a challenge. Understanding is hampered by the relative lack of driver mutations and the massive genetic instability that characterises many forms of the disease and makes molecular profiling difficult.

Genomic and transcriptomic studies have managed to identify a limited number of driver mutations, restricted at present largely to p53 and BRCA1/2, but have grossly failed to identify predictive patterns that can be used to determine therapy. A notable exception is the identification of defective homologous recombination as a predictor of response to both platinum [1] and PARP inhibitor therapy [2].Building upon this, and using defects in other DNA damage repair (DDR) mechanisms as a stratification tool, has enormous potential. The inability to repair DNA damage is one of the hallmarks of the cancer cell, however rendering a cell defective in DNA damage repair is not mediated through a single process, rather it is conferred by a wide variety of genomic defects and this can result in differing sensitivities to conventional and novel chemotherapeutic agents.

**Fig. 1:**
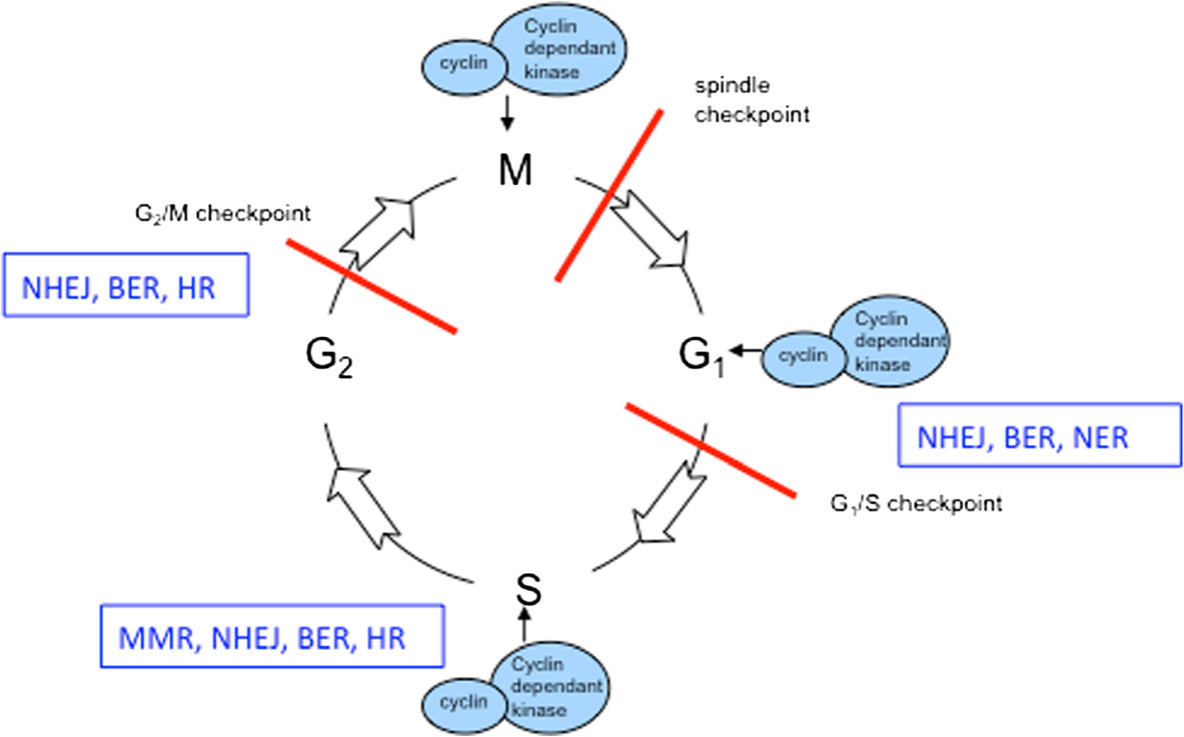
The phases of the cell cycle and DNA repair mechanisms active during the four stages. Both classical and alternative non homologous end joining (C-NHEJ and A-NHEJ) repair double strand breaks and are active throughout the cell cycle, they are especially active in G1 [25]. Base excision repair (BER) removes damaged nucleotides and repairs single strand breaks throughout the cell cycle [60]. Nucleotide excision repair is concerned with the removal of bulky damaged DNA lesions and single strand breaks during G1. Mismatch repair corrects DNA mismatches and repairs single strand breaks during S phase [91]. Homologous recombination repairs double strand breaks during S phase and G2 [4]

In general, DNA damage results in either single strand or double strand breaks. Current understanding suggests that there are five mechanisms used by mammalian cells to identify and repair DNA damage. These are mismatch repair (MMR), base excision repair (BER), and nucleotide excision repair (NER) for single strand breaks and homologous recombination (HR) and non homologous end joining (NHEJ) for double strand breaks (see Fig. 1). Single strand breaks may be repaired directly but if left unrepaired will result in a stalled replication fork. Replication stress is the term coined to describe the effects on the cell of a stalled replication fork. Replication stress should activate functional DNA checkpoint proteins to trigger the commencement of the double strand DNA repair pathways. However, in cancer cells the combination of a high replication rate and defective DNA repair mechanisms can lead instead to genomic instability. This is particularly the case in high grade serous ovarian cancer which is characterised by genomic instability. Given the high levels of genomic instability and subsequent chromosomal rearrangements which are tolerated by high grade serous cancer cells in particular, it is highly likely that at least one of these five pathways is dysfunctional in each cell (Table 1).

**Table 1:** The known contribution of defective DNA repair pathways to ovarian cancer

DNA repair pathway	Type of repair	Contribution to serous ovarian cancer	Contribution to all histological subtypes of ovarian cancer
Homologous Recombination	Double Strand Breaks	50% [4]	50%
Non Homologous Enjoining	Double Strand Breaks		
Base Excision Repair	Nucleotide Excision and Single Strand Breaks		Polymorphisms and altered expression of BER components in 63–67% ovarian cancers [60, 91]
Mismatch Repair	Single Strand Breaks, Nucleotide Mismatches	0.18% [94]	0.76% [94], mostly clear cell
Nucleotide Excision Repair	Single Strand Breaks, Bulky Lesions	8%	

Through the understanding of these DNA repair mechanisms, their interaction and how they may be defective in women with different subtypes of EOC, it may be possible to target chemotherapy to the molecular mechanisms underlying a cancer. Synthetic lethality is the term coined to describe the effect of the combination of two genetic alterations that are lethal to cells, but alone do not have a negative effect (reviewed by Kaelin [3]). Discovery of novel drugs which exploit synthetic lethality is one such way that the understanding of defective DNA repair mechanisms can advance the management of ovarian cancer.

Treatments exploit the exquisite balance of DNA damage in a cancer cell by either inducing further DNA damage, with which the cell’s already impaired DNA capacity cannot cope, or by impairing the capacity of the DDR even further. Either strategy leads to catastrophic DNA damage causing cell death. The benefit of the latter approach in which DDR pathways are inhibited in the cell, is that it has little effect on normal cells which include many overlapping pathways that can cope with loss of a single pathway. Thus the toxicity of these targeted treatments is often more acceptable than conventional cytotoxic chemotherapy.

Here we review the current state of knowledge regarding the role of the various DNA repair pathways in ovarian cancer; using high grade serous cancer as the exemplar, but referring to the other subtypes where appropriate.

### Homologous recombination

#### Mechanism

Homologous recombination (HR) is a high fidelity mechanism for repairing double strand breaks or stalled replication forks that occur during S and G2 phases of the cell cycle [4]. DSBs can be caused by ionising radiation, reactive oxygen species and antineoplastic drugs, such as anthracyclines and bleomycin. Inability to repair DSB causes chromosomal rearrangements and can lead to cell death [4].

**Fig. 2:**
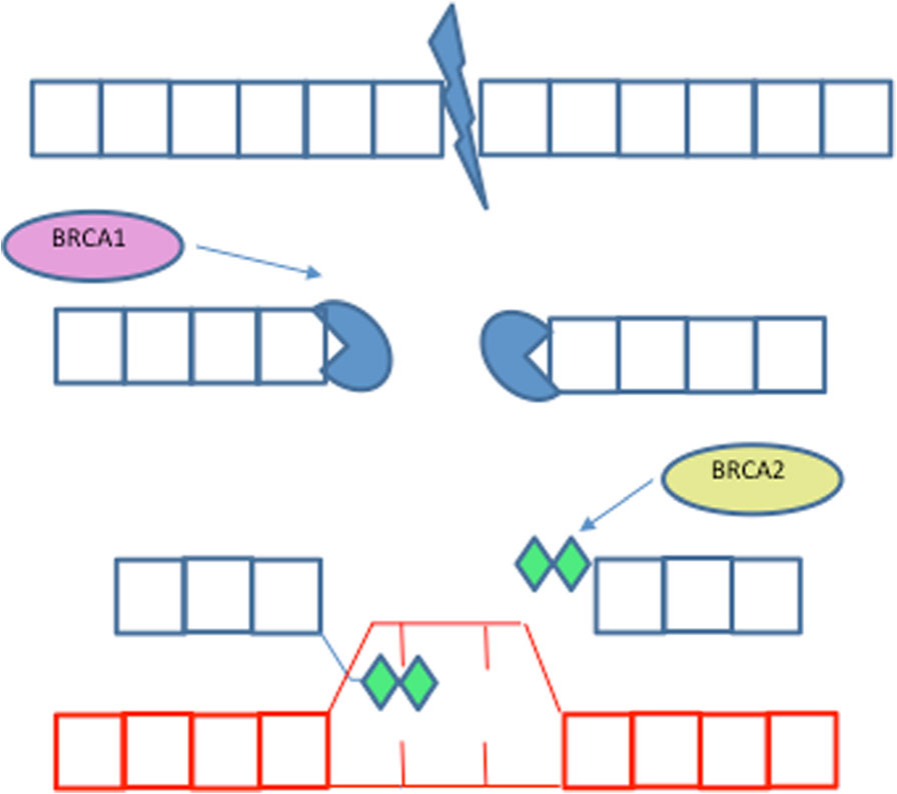
Double Strand Break (DSB) repair by Homologous Recombination. When a DSB is recognised, BRCA1 mediates the recruitment of the MRN complex, which comprises MRE11, RAD50 and NBS1 [3]. The MRN complex recruits ataxia-telangiectasia mutated (ATM) which, in turn, activates MRN components by phosphorylating them. The MRN complex work with CtIP and EXO1 to resect 3′ ends of the DS. Replication protein A (RPA) covers the DNA overhang to prevent it from being degraded. This stage of HR, nucleolytic processing, can be defunct in hereditary ovarian cancer; a single base mutation in exon 10 of MRE11 is reported in hereditary ovarian cancers [92] and RAD50 protein truncation is reported in hereditary breast and ovarian cancer families. As well as activating nucleolytic processing machinery, ATM phosphorylates and activates BRCA1 and RAD51. ATM is mutated in 2% of high grade EOC [7] and is significantly associated with platinum resistance [41]. Single strand DNA is unwound and the damaged chromosome invades the sister chromosome to form a Holliday junction. This occurs when RPA is replaced by RAD51, facilitated by BRCA2, allowing for strand invasion [93]. RAD51 is hypermethylated in 3% of high grade serous EOC, resulting in ineffective strand invasion [7]. MSY (not shown) is an oncogene that can silence the activation domain of BRCA2 it is overexpressed in 17% sporadic high grade serous ovarian cancer [15]

HR uses the sister chromatid as a template to repair the DSB. The damaged DNA ends of the DSB are resected by the RAD50, MRE11 and NBS1 complex (mediated by BRCA1). The complex, using a 3′-5′ exonuclease exposes the 3′ strand ends on either side of the DSB [5]. Single strand DNA is unwound and the 3′ strand from the damaged chromosome invades into the sister chromosome, at the complementary sequence, using BRCA2 and the single strand binding protein RAD51. DNA polymerase then reads off the complimentary sequence to extend the previously damaged 3′ end. The replication continues past the original DSB and continues to the end of the chromosome (see Fig. 2) [6].

It has been estimated that there are at least 40 proteins participating in this pathway, but it was the recognition that this included BRCA1 and BRCA2 that provided the stimulus to study this pathway in breast and ovarian cancer.

### The role of homologous recombination in high grade serous ovarian Cancer

Overall, defects in HR, whether through mutation or epigenetic silencing of key proteins, have been found in up to 50% EOC [1, 7].

### BRCA1 and 2

Mutations in BRCA1 and BRCA2 are well documented in hereditary ovarian cancer syndromes. As a result of carrying mutations in BRCA1 and BRCA2 women carry a 40% lifetime risk of epithelial ovarian cancer [8].

BRCA1 and 2 dysfunction is also prevalent in sporadic epithelial ovarian cancer [9]. The term “BRCAness”, which is synonymous with defective homologous recombination (HRD), has been coined to describe the phenotype of dysfunctional BRCA1 and 2, brought about by epigenetic silencing. Mechanisms contributing to HRD in sporadic EOC include BRCA1 hypermethylation, occurring in 5–31% of sporadic ovarian cancer [10, 11]. Estimates of the prevalence of BRCA1 and 2 mutations in the general population range from 1 in 400 to 1 in 800 [12].

Of the histological subtypes of EOC, high grade serous EOC is associated with a BRCA1/2 mutations of 10.9 and 5.6% respectively, whilst endometrioid EOC is associated with a 2.1% mutation rate [13]. BRCA1 and 2 do not appear to play a role in mucinous EOC.

### Implications for therapy

Defective HR allows accumulation of mutations thereby promoting oncogenesis, but appears to also confer a prognostic advantage, possibly through improved sensitivity to chemotherapeutic agents and ionising radiation [14, 15].

This effect is not limited just to BRCA related cancers and it is now clear that all HR defective tumours, identified using a RAD51 immunoflourescence assay, are associated with improved survival compared to HR competent tumours when managed with platinum based chemotherapy [1].

Therefore, disruption of functional HR is a potential therapeutic target to prevent chemotherapy resistance. A clinical example, although with little current clinical to support it, is the use of the BCR-ABL inhibitor, Imatinib. RAD51 requires phosphorylation in order to become active during the process of homologous recombination and phosphorylation is dependent on the proto-oncogene ABL1. Thereby using Imatinib, HR is inhibited and cells are sensitised to crosslinking chemotherapy agents and ionising radiation [16].

### PARP inhibitors

Since the landmark papers of Bryant and Farmer [17, 18], the therapeutic effect of PARP inhibition has been presumed to be mediated via the inhibition of the base excision repair pathway in cells already defective in homologous recombination. The concept of HR/BER synthetic lethality is based upon the idea that most single strand breaks would be repaired by BER, if this is inhibited by PARPi then they persist to become stalled replication forks during replication which then cannot be repaired by the defective HR in the cell.

PARP inhibitors have shown clinical efficacy in tumours with defective HR. The PARPi, olaparib, has shown efficacy in both the relapsed setting, in women with BRCA1/2 mutations and ovarian cancer [19], and when used as maintenance therapy in women with sporadic high grade serous ovarian cancer, improving progression-free survival time, when compared to placebo [20]. However the remaining challenge is to identify HR defective tumours; in a recent phase III study of niraparib, which demonstrated efficacy in women with platinum sensitive relapse, the greatest effect was, not surprisingly, seen in tumours with a germline BRCA mutation but the test used to determine HR status in the non BRCA related tumours failed to provide a clinically useful stratification [21].

### Other HR related targets

Whilst PARP inhibitors have clear clinical utility and serve as a great example of synthetic lethality, there are other opportunities to target HR related proteins including ATR.

ATR is a potential target as it works upstream in the HR process and is influential over the whole pathway. ATR initiates HR in response to radiation or platinum induced stalled replication forks and ATR inhibition should render the cell defective for HR [22]. NU6027 has been shown to inhibit ATR in preclinical studies and has been found to enhance hydroxyurea and cisplatin cytotoxicity, inhibit RAD51 focus formation, and lead to cell cycle arrest at G2/M. Further, when used with PARPi or cells with defective XRCC1, NU6027 demonstrates synthetic lethality [23].Two ATR inhibitors, VX-970 (similar to VE-821) and AZD-6738, are currently undergoing clinical evaluation. VX-970 is being evaluated both as a single agent and in combination with platinum-based chemotherapy (clinicaltrials.gov identifier: NCT02157792) and AZD-6738 in haematological malignancies with 11q deletions (ATM defective) (clinicaltrials.gov identifier: NCT01955668).

A further way to block HR is to inhibit CDK1, which activates BRCA1. Preclinical studies showed that CDK1 inhibitor AG024322 is synthetically lethal with PARPi [24].

### Non homologous End Joining

#### Mechanism

Non homologous end joining (NHEJ), the other pathway in the repair of DNA DSBs, has received less attention than HR as a determinant of response to therapy. Emerging evidence suggests that this may be a serious oversight and that NHEJ may have a major role in determining outcome to treatment. NHEJ occurs during G0 and G1 of the cell cycle, with some activity in late S and G2 [25] and is considered to be responsible for the majority of DSB repairs caused by ionising radiation [26].

NHEJ has always been considered to be an error prone process relying on modification of DNA breaks to allow joining (or synapsis), rather than directly replicating the sister chromatid [27]. Whilst it is not entirely clear why cells choose one pathway over the other it has been hypothesised that higher eukaryotes use the error prone NHEJ more than HR because NHEJ allows for faster DNA repair during a dynamic cell cycle and does not have the same steric constraints as HR, where the sister chromatid needs to be in close proximity [25, 28]. The NHEJ process comprises two pathways, a classical and an alternative pathway [29].

#### The classical NHEJ pathway

The classical, canonical, or DNA-PK dependent pathway, is active in normally functioning cells and occurs in the absence of a DNA template or extended regions of microhomology. The classical pathway is inherently accurate but is adaptable and therefore infidelity can creep into the process as a result of the nature of the damage that requires repair [30].

Classical NHEJ involves the actions of 6 key proteins: DNA-PKcs, the Ku70/Ku80 heterodimer, Artemis, DNA ligase IV, XRCC4 and XLF [29].

Ku (Ku70/Ku80 heterodimer), a ring like structure, binds to the DNA DSB with high affinity and recruits the serine-threonine protein kinase DNA-PK. Ku is able to stabilise DNA-PK’s affinity for the DNA terminus [31] and may act as a docking site for other proteins in NHEJ. If the ends of the DNA DSB are not compatible then ‘overhangs’ of DNA need to be removed, trimmed back to a microhomology and resynthesized by nucleases and DNA polymerases such as Artemis. Artemis can form both a 3′ and a 5′ endonuclease when complexed with DNA-PK [32]. Once the ends are compatible they can be ligated. DNA ligase IV and XRCC4 co-factor, along with XLF appear to be responsible in humans for this ligation in NHEJ [33, 34].

#### The alternative NHEJ (A-EJ) pathway

The alternative, (also known as the back up or microhomology mediated end joining) pathway is less well characterised than the classical pathway. As the name suggests, it has been found to be active when other mechanisms, (classical NHEJ and HR) are inhibited or absent. It can be activated throughout the cell cycle and is slower than the classical pathway [35]. A-EJ pathway is independent of Ku and frequently uses microhomologies distant from the DSB. A-EJ is typically associated with deletions at the repair junction and therefore is highly mutagenic. The key proteins proposed to be active in the alternative pathway are PARP1, DNA ligase III and XRCC1 and thought to be promoted by DNA polymerase Θ [36]. PARP1 recognises and binds to DNA at DSB in competition with Ku to fulfil a similar role [37]. XRCC1 and LIG III form a complex to ligate the broken DSB strands [38].

Ku is biochemically more competitive than PARP1, however in cells that are Ku80 deficient, inhibiting PARP results in both arms of NHEJ being defective, resulting in the cell being sensitive to the effects of ionising radiation [39].

#### Role in high grade serous epithelial ovarian Cancer

Errors in NHEJ are associated with therapeutic resistance. Defective NHEJ has been found in up to 50% of ovarian cancers, is independent of HR function and appears to confer resistance to PARP inhibitor therapy, at least in the ex vivo setting [40]. Defective NHEJ may be conferred by a wide range of molecular aberrations affecting either the classical or the alternative pathways. In general, molecular events can either be in the germline, conferring an altered risk of developing the disease, or somatic, implying that the genomic event is affecting cell survival and subsequent sensitivity to therapy.

#### Classical pathway components

##### DNA-PK complex

DNA-PK is the complex made up of DNA-PKcs, Ku70/Ku80. Molecular study of platinum resistant ovarian cancer has shown that DNA-PK is responsible for phosphorylating AKT at S743 and relocating it to the nucleus. High expression of DNA-PKCS is a common finding in high grade serous cancer and associates with advanced stage, higher grade and poorer survival due to reduced platinum sensitivity [41]. PARP-1 has been shown to interact with Ku protein [42]. Genetic ablation of KU70 and LIG IV has been shown to restore survival of PARP-1 deficient cells exposed to DSBs inducing agents [43]. Also, DNA-PK inhibition and depletion has been shown to result in HR function recovery and PARPi resistance in vitro [44].

#### DNA LIG iv

Single nucleotide polymorphisms have been associated with an increased risk of developing ovarian cancer [45] although this remains controversial [46]. It is possible that LIG IV variants result in dysfunctional NHEJ with an associated hypersensitivity to DNA damage [33].

#### Alternative pathway components

##### Pol Θ

Pol Θ is thought to promote A-EJ. Pol Θ expression has been found to be inversely proportional to HR competency in epithelial ovarian cells in vitro and increased expression has been found in serous ovarian cancer [47].

##### XRCC1

Although often thought of as a protein whose role is limited to NER, XRCC1 is relatively promiscuous and appears to have functional roles in both the base excision repair (BER) and non homologous end joining pathways. Deficiency in XRCC1 can result in cell death due to genomic stress, but can also allow for accumulation of mutations, as SSBs remain unrepaired. In ovarian cancer patients XRCC1 polymorphisms are associated with poorer prognosis and higher levels of platinum resistance [48].

Pre-clinical studies of cells deficient in XRCC1 show that these cells have higher sensitivity to platinum chemotherapy than controls [49]. This is supported by clinical data suggesting that EOC tumours overexpressing XRCC1 and were more likely to be high grade serous cancer, demonstrate platinum resistance, increased risk of death and increased risk of tumour progression [49].

### Implications for therapy

#### PARP inhibitors

Knowledge of NHEJ status may allow further enrichment of a population that is likely to have a high response rate to PARPi therapy.

DSB are repaired by the dominant, but more error prone NHEJ pathway. Only if cells are NHEJ defective (classical pathway) will DSB will be repaired by HR. Recent data suggest that cells which are NHEJ defective develop upregulated HR, as evidenced by increased RAD51 foci formation and are resistant to rucaparib [40]. However cells which are HR defective are still sensitive to PARPi, as the alternative, PARP dependent pathway is also inhibited.

#### DNA-PK

It is now becoming clear that platinum resistant clones may be present in the primary tumour and can be selected for, inadvertently, through the use of platinum based chemotherapy [50]. Platinum resistant high grade serous EOC are associated with phosphorylated AKT, relocated to the nucleus. By inhibiting DNA-PK or inhibiting AKT, platinum sensitivity can be restored in cells rendered previously resistant [51].

Preclinical studies have found that treatment of malignant ovarian cell lines with the combination of an EGFR inhibitor, Gefitinib, plus cisplatin is able to reduce DNA-PK expression, even in cisplatin resistant cells [52]. Cisplatin has also been reported to inhibit NHEJ in vitro [53]. Unravelling intracellular signalling properties of already established drugs may be a rewarding avenue in the future management of high grade serous ovarian cancer.

The finding that Wortmannin, a member of the DNA-PK family which inhibits PIKK, and is a radiosensitiser in preclinical models, [54] led to the investigation of other DNA-PK inhibitors as potential targets such as Nu7441, a small molecule DNA-PK inhibitor found to sensitise HeLa cells to ionising radiation and etoposide [55, 56]in colon cancer. There are other small molecular DNA-PK inhibitors, however none are yet reported to have shown activity to high grade serous ovarian cancer.

#### XRCC1

XRCC1 expression has been suggested as a predictive biomarker in human ovarian cancer with expression being associated with platinum resistance, suggesting that XRCC1 positive tumours should be considered for non platinum based chemotherapy [49].

Furthermore, XRCC1 may be a target for synthetic lethality, it is active in multiple DNA repair pathways, with important activity in single strand repair pathways, meaning that cells deficient in XRCC1 have higher reliance on DSB repair pathways to maintain genomic integrity. Pre-clinical data, albeit regarding breast cancer, has examined the synthetic lethality of inhibiting pathways for DSB in cells deficient in XRCC1 and found that potent ATM and DNA-PKc inhibitors are synthetically lethal in XRCC1 deficient cells [57].

### Base excision repair

#### Mechanism of base excision repair

Base excision repair (BER) is responsible for both removing small base lesions from DNA and also effecting SSB repair (via a subpathway of BER called single strand break repair (SSBR)). Bases may become damaged due to reactive oxygen species (ROS), spontaneous deamination or due to alkylation [58]. Single strand breaks (SSBs) are the commonest endogenous DNA lesion. They arise from DNA damage due to reactive oxygen species (ROS) or via BER when damaged bases are excised [59].

BER repairs lesions generated endogenously by aerobic respiration, but also therapeutically by ionising radiation, topoisomerase I agents and DNA methylating agents.

**Fig. 3:**
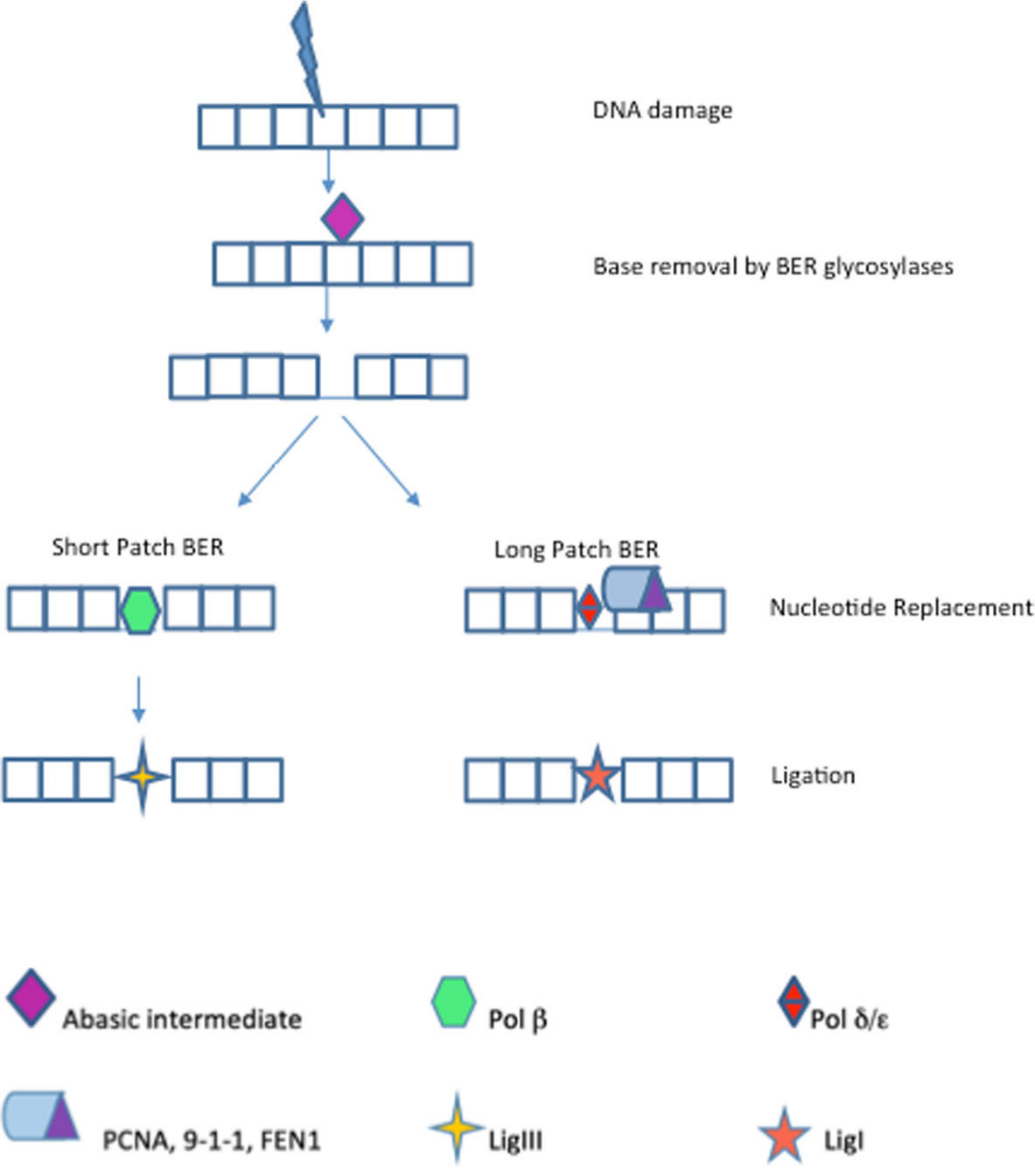
The Base Excision Repair Pathway. BER consists of 5 stages: Damaged bases are recognised and removed by BER glycolases to produce an abasic intermediate. BER endonucleases (commonly APE1) make a site incision, generating a SSB. The sugar backbone is modified by phosphodiesterase. The newly formed nucleotide gap is filled by DNA polymerase. DNA ligase seals the nucleotide patch together. In SP-BER DNA polymerase B (Pol β) replaces the damaged nucleotide and the ends are ligated by DNA Lig III [58]. In LP-BER the patch is processed by proliferating cell nuclear antigen (PCNA), 9–1-1 complex and flap endonuclease 1 (FEN1), the nucleotides replaced by Pol δ and Pol ε with ligation by Lig I. in the single strand break repair (SSBR) pathway, PARP1 senses nicks in DNA, recruits XRCC1which acts as a scaffold for the other BER components to stabilise the SSB

BER can work by repairing one nucleotide in the predominant, short patch BER (SP-BER)pathway or by replacing 2–12 nucleotides as a long patch BER (LP-BER) [58] (Fig. 3).

### Role in high grade serous epithelial ovarian Cancer

The extent to which defective BER plays a role in high grade serous epithelial ovarian cancer is not well identified, probably because of the difficulty of studying this pathway independently of other DNA repair pathways. However, there are some studies identifying how the components of BER may contribute to ovarian cancer.

Polymorphisms of other BER components, notably the glycosidase OGG1, result in an increased risk of ovarian cancer, especially in women who are BRCA1 deficient [60], and are a common finding with up to 63% of women with ovarian cancer carrying these germline variants [61].

DNA polymerase activity upregulates the error prone BER pathway, via pol β overexpression, in up to 67% of ovarian cancers. Subsequent silencing of pol β induces sensitivity to platinum chemotherapy, thus implicating pol β in the low fidelity DNA repair mechanism which allows cells to escape apoptosis by bypassing DNA lesions [62].

High nuclear expression of APE1 is associated with high grade serous epithelial ovarian cancer and correlates with poor surgical outcome, a poor overall survival and possibly higher rates of platinum resistance when compared to APE1 negative patients [63].

### Implications for therapy

BER has the capacity to repair DNA which has been intentionally damaged by therapy such as IR and alkylating agents. Therefore, by undermining BER whilst managing patients with IR and chemotherapy, the effects can be potentiated.

#### PARP inhibitors

PARP recruits and activates DNA repair proteins by causing the post translational modification, poly ADP-ribosylation. PARP1 has a key role in SSBR, as it binds at single strand breaks, stabilising the DNA ends and recruiting downstream proteins such as XRCC1. Inhibition of PARP has been found to sensitize ovarian cancer cells to chemotherapy because when PARP is inhibited, BER cannot proceed and the SSB causes replication fork collapse when it is met in S phase, resulting in DSB [64, 65], If the DSB cannot be repaired, then the cell dies. As previously mentioned, PARP inhibition is synthetically lethal in women with known defective homologous recombination. Olaparib is being investigated in clinical trials, not only as a monotherapy (clinicaltrials.gov identifier: NCT01844986) but also in combination with AZD1775, a cell cycle checkpoint inhibitor (clinicaltrial.gov identifier NCT02511795), [66].

#### Pol β

Pol β has been identified as a potential target for inhibition which would result in abrogation of BER and prevent the tumour cell from withstanding oncogenic stress. One inhibitor, masticadienomic acid (MA) has been identified as a potent and selective Pol β inhibitor, which competes for the active site of Pol β in ovarian cancer cell lines and sensitises the cells to cis-platin [67]. Additional in vitro studies have found Pol β inhibitors able to cause sensitisation to bleomycin and ionising radiation [68, 69].

#### APE1 inhibitors (APEX1)

In vitro studies of silencing APE1 using siRNA rendered ovarian cancer cell lines sensitive to cisplatin and resulted in cell apoptosis [70].Methoxyamine has been found to inhibit APE1 in other cancers and studies of methoxyamine in ovarian cancer cell lines have found it to increase sensitivity to alkylatortemozolmide [71, 72]. There are multiple ongoing clinical trials of methoxyamine in various advanced or relapsed solid tumours, including ovarian cancer (clinicaltrials.gov identifiers NCT02535312, NCT01851369).

### Mismatch repair

#### Mechanism of mismatch repair

Mismatch repair (MMR) is a mechanism for repairing incorrect base matches and insertion/deletion errors generated during replication. Defects in MMR are associated with genome instability due to higher rates of spontaneous mutation and thus high levels of microsatellite instability (MSI). MMR plays an instrumental role in DNA repair but is also required for effective cell cycle arrest and apoptosis in severely damaged cells [73]. Hence, defective MMR results in a predisposition to certain cancers and a loss of sensitivity to chemotherapeutic agents.

**Fig. 4:**
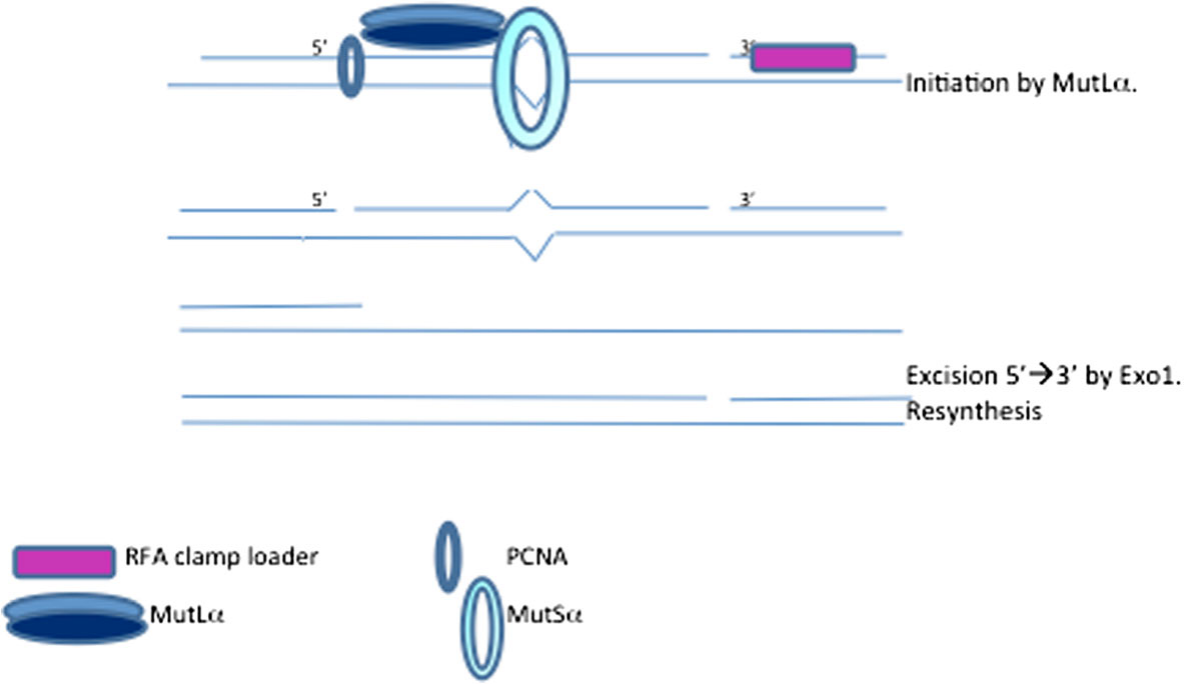
Eukaryotic mismatch repair mechanism. DNA mismatches are recognised by the mismatch recognition factor MutSα, which then prompts binding and DNA stabilisation by the MLH1-PMS2 heterodimer (MutLα). The PCNA and RFA clamp loader are able to load on at a nearby ‘nick’. MutSα activated exonuclease 1 (Exo1) excise the mismatch in a 5′ to 3′ direction and the excised fragment of DNA is removed by the DNA polymerase δ holoenzyme. DNA polymerase δ refills the DNA gap and it is ligated by DNA ligase I

MMR has been extensively studied in E.coli, but is incompletely defined in eukaryotic cells. MMR comprises 3 general stages, initiation, excision and resynthesis (Fig. 4).

MutS homologs (MSK2, MSH3, MSH6) recognise and bind to mismatches and loops caused by the insertion/deletion of up to 4 bases to form the hMutα or hMutβ heterodimer complex. This occurs as hMutLα stabilises the bind and distinguishes the newly formed, mutated, DNA by scanning DNA for SSB or ‘nicks’. Nicks are present transiently in newly synthesised lagging strand DNA, before DNA ligase seals them, so nicks are the eukaryotic method for distinguishing new DNA from template DNA. The next stages are incompletely understood in eukaryotes, but overall, DNA is then unwound by a helicase and excised by exonuclease I (Exo I), this is followed by resynthesis by polymeraseδ and ligation by DNA ligase I, with the aid of the DNA sliding clamp, proliferating cell nuclear antigen (PCNA).

Induction of cell apoptosis using MMR machinery is hypothesised to happen by one of two possible mechanisms (futile repair mechanism or the direct signalling model), which both result in the recruitment of AMT/ATR by hMutSα and hMutLα.

#### Role of mismatch repair in high grade serous epithelial ovarian cancer

Microsatellite instability occurs when there are variable lengths of unrepaired nucleotide repeats, MSI is used as a marker for defective MMR in studies to determine the frequency and contribution of defective MMR to cancer development.

Defective MMR is understood to be a driver in Lynch Syndrome, an autosomal dominant syndrome where individuals are predisposed to colorectal, endometrial and ovarian cancers. The study of Lynch Syndrome, like other hereditary cancer syndromes, has allowed a better understanding of the role of MMR proteins in cancer (Table 2). Lynch Syndrome confers a 10–15% lifetime ovarian cancer risk, with ovarian cancer occurring at a younger age. In contrast to BRCA1 and 2 mutations, the Lynch Syndrome phenotype predisposes to low grade [74] or clear cell histology [75]. A large study of 2222 ovarian cancer cases found that defective MMR contributed to only 0.18% cases of high grade serous ovarian cancer [76].

**Table 2:** Defective MMR proteins in ovarian cancer

Gene/Protein	Role	Defect	Clinical Effect	Evidence of mutation in cases of HGS
MLH1	Forms MutLα with PMS2 [95] which has endonuclease activity.	Promoter methylation	Spontaneous tumours	0.1%
		Exon deletion	Lynch Syndrome	
MSH6	Forms hMutSβ with MSH2 to recognise and initiate repair [95]	Exon deletion	Lynch Syndrome	0.45%
MSH2	Forms hMutSβ			0.18%
PMS2	Forms MutLα	Loss		0.05%
MutLα and MutSβ	Recruit ATM/ATR	Loss	Resistance to DNA methylating agents [77]	

MMR is defective in sporadic ovarian cancer also, where promotor methylation results in epigenetic silencing of key genes (Table 2). The main proteins defective in Lynch Syndrome are MHLH1 and MSH6.

#### Implications for therapy

In vitro studies have found an association between MMR deficiency and resistance to platinum agents and DNA methylating agents such as 6-thioguanine, due to an inactivation of MMR driven apoptosis in ovarian cancer cell lines [77, 78]. The extent to which defective MMR contributes to therapy resistance in vivo, however, is inconclusive [79] for high grade serous ovarian cancer, but is better defined in ovarian clear cell carcinoma where defective MMR plays a larger role in oncogenesis [80]. Nonetheless, reactivation of MMR by demethylation of epigenetically silenced MMR genes is an interesting area of study. In vitro and mice studies, 5-azacytidine and 2′-deoxy-5-azacytidine (DAC or decitabine) have been found to reactivate MMR by demethylating the mMLH1 promoter, resulting in the return of sensitivity to platinum therapies, temozolomide and epirubicin [81, 82]. However, phase II clinical trials using decitabine with carboplatin in ovarian cancer were abandoned due to a lack of efficacy and the development of adverse reactions (DACROC study).

### Nucleotide excision repair

#### Mechanism of nucleotide excision repair

Nucleotide excision repair (NER) is a highly conserved and versatile pathway used to remove ‘bulky lesions’ caused by UV light and carcinogens such as benzopyrene, which would otherwise distort the DNA double helix. Large protein complexes scan the DNA and identify distortions at the bulky lesion. Lesions that stall transcription use the ‘transcription coupled NER pathway’ using RNA Pol II. The ‘global NER pathway’ manages lesions elsewhere affecting the genome. The transcription factor, TF11H, has helicase activity and is recruited to unwind the DNA duplex. XPG and ERCC1-XPF then cleave the abnormal strand either side of the lesion and DNA polymeraseδ or ε and ligase 3(LIG III) repair the gap with PCNA and replication factors C [59].

Defective NER is implicated in various cancers, notably the syndrome xerodermapigmentosa which is characterised by UV sensitivity, where global NER is defective. Epigenetic alterations, gene polymorphisms and SNP’s of global NER proteins are associated with lung, skin and bladder cancer.

#### Role of NER in high grade serous ovarian cancer

NER is responsible for repairing intrastrand DNA cross links caused by platinum therapy and has been found to determine platinum resistance in ovarian cancer cells in vitro [83, 84]. The TCGA data set analysis revealed that 8% of high-grade serous EOCs have an NER gene alternation which included homozygous deletions of NER genes and nonsynonymous or splice site mutations. The extent to which polymorphisms of NER genes and their subsequent protein expression plays a role in the platinum resistance of ovarian cancer is controversial, with some studies finding direct correlation between protein levels to platinum resistance and other studies finding no correlation, (see Table 3) [85, 86]. However, Ceccaldi et al.’s extensive TCGA study demonstrated that NER gene alterations in high grade serous EOCs were associated improved overall and progression-free survival compared to tumours that did not have NER alterations. Specifically, two NER mutations; ERCC6-Q524 and ERCC4-A583T were functionally associated with platinum sensitivity in vitro.

**Table 3:** NER components and response to platinum therapy in ovarian cancer

Protein	Role	Relevant to Ovarian Cancer
ERCC1	Forms heterodimer with ERCC4 to form XPF, which binds DNA with nuclease activity	SNP C8092A, which affects mRNA stability associated with reduced tumour size and more differentiation [96].
		In vitro cell line experiments found high ERCC1 expression correlated to platinum resistance due to upregulated NER [97]
XPA	Binds and stabilises damaged DNA	Paradoxically XPA expression associated with better overall survival in women with metastatic ovarian cancer and chemotherapy response [98]
XPB	A transcription factor that can also aid unwinding of DNA at sites of damage	Five fold higher mRNA expression in platinum resistant ovarian cancer cell lines than non resistant cells [89].
XPG	Endonuclease that catalyses 3′ incision	No statistically significant effect of XPG polymorphisms [94]
CSB	ATP dependent helicase that enables DNA repair	Six times higher mRNA expression in platinum resistant ovarian cancer cells [98]

In the same study the survival of patients with tumours harbouring NER alterations was similar to patients with *BRCA1/2*-altered tumours suggesting that NER pathway alterations may contribute to EOC platinum sensitivity to a similar extend as that of the effect of *BRCA1/2* loss. Ceccaldi’s data reported that NER pathway alterations, unlike *BRCA1/2* alterations, were not associated with sensitivity to PARP inhibitors. Importantly, their findings identified a subset of NER-deficient, HR-competent EOCs with distinct platinum and PARP-inhibitor profiles [87].

An interesting study by Wang et al. looked at the roles of both NER and HRR in platinum resistance. They found that the importance of NER’s contribution to platinum resistance varies between in vitro cell lines. Additionally, overall there was no correlation between NER protein component expression and NER activity, nor cisplatin sensitivity [88]. Yet, they did highlight that ERCC1, the role of which is extensively studied in NER and platinum resistance, also plays an influential role in HRR. Delineation of the NER and HRR pathways in these cells will be important when developing DNA repair pathway modulators.

#### Implications for therapy

Development of NER inhibitors is a potential target for the management of platinum resistant ovarian cancer. There are a number of small molecular inhibitors, identified through in silico screens, with variable clinical potential.

One promising agent is Trabectedin, which is thought to disrupt the NER pathway by binding NER machinery and DNA at the damaged DNA site, sequestering NER proteins and resulting in cytotoxic complexes [83, 89]. The INOVATYON study is a phase III trial investigating Trabectedin and pegylated liposomal doxorubicin (PLD) vs carboplatin and PLD in women with platinum resistant ovarian cancer(clinicaltrials.gov identifier NCT01379989).

### The importance of developing a DDR stratification system

Targeting functional and dysfunctional DNA repair pathways in ovarian cancer, to overcome therapeutic resistance is a frontier for improving the prognosis of ovarian cancer. However multiple challenges exist. Firstly, clinicians require a way of identifying which DNA pathways are important in a particular tumour. Secondly, an understanding of the interactions and overlap between the pathways will be key to accurately targeting therapy. Thirdly, although there are numerous small molecular DNA repair pathway modulators which appear promising at a pre-clinical stage these need to be rapidly translated through clinical development.

The first two issues may be overcome through the development and use of functional assays to either identify key DNA repair pathways or identify key DNA repair proteins (‘key drivers’), which may interact across numerous pathways.

Importantly however, determining the DDR status of any given HGSOC cancer is not trivial. Despite multiple attempts to an assay for HR an ideal test remains elusive. While BRCA-mutated cancers display a distinctive gene expression signature, this in itself is not necessarily predictive. In a recent study, 11 out of 12 tumours with dominant BRCA signatures were refractory to primary treatment, consistent with intact HR [84]. Furthermore, attempts to use indirect methods such as genomic scarring have been equally ineffective within the context of clinical trials [21].

Studies to identify HR proficiency using the identification of Rad51 focus formation in primary cultures, gathered from ascitic fluid in women with ovarian cancer have shown a promising way of identifying women with HR deficiency who are susceptible to PARP inhibition [85]. Similar functional assays have been developed for the assessment of NHEJ and NER status [86]. Development of these assays further, for clinical use, may prove useful in selecting women for targeted therapy via identification of their DNA repair status.

If we know that a drug targets a specific protein in a specific DNA repair pathway, then assessing function of that protein represents an important biomarker. This can be carried out by assessing the DNA, RNA or protein directly. Immunohistochemistry of tissue samples can be used to screen for key proteins and stratify patients into groups likely to respond to the drug. Early work using immunohistochemistry and RT-PCR to identify DNA-PK/mRNA in solid tissue as a predictor for NHEJ and correlation with sensitivity to PARPi and cisplatin sensitivity is one such promising example of this [90].

## Conclusions

With the increasing realisation that ovarian cancer is not one disease, the increased use of molecular diagnostics, and the development of new targeted agents the ability to deliver personalised medicine for patients with ovarian cancer is perhaps finally on the horizon.

An understanding of the DNA damage repair pathways and how they are abrogated will allow the development of personalised treatment algorithms but as we have outlined here this is likely to be complex given the high degree of interaction, overlap and perhaps redundancy that is shown between the various pathways.
